# Adaptive physiological and biochemical response of sugarcane genotypes to high-temperature stress

**DOI:** 10.1007/s40502-018-0363-y

**Published:** 2018-04-12

**Authors:** S. Kohila, R. Gomathi

**Affiliations:** Plant Physiology, ICAR-Sugarcane Breeding Institute, Coimbatore, Tamilnadu 641 007 India

**Keywords:** Elevated temperature, Chlorophyll, F_v_/F_m_ ratio, Antioxidant enzyme, Sucrose metabolizing enzymes, Leaf gas exchange

## Abstract

Impact of elevated temperature on physiological and biochemical changes were evaluated in 5 commercial sugarcane genotypes and 2 wild species clones at two different growth phases. The study revealed that heat stress decreased chlorophyll content, chlorophyll stability index (CSI), SPAD value, maximum quantum efficiency of PSII photochemistry (F_v_/F_m_ ratio), leaf gas exchange parameters, relative water content (RWC), and activities of nitrate reductase (NR), sucrose-metabolizing enzymes (SPS, SS, AI, NI) in all the genotypes and species clones. In contrast, elevated temperature induced an increase in proline, total phenolics content (TP), antioxidant enzyme activities (SOD and POX), lipid peroxidation (LP), membrane injury index (MII) and soluble sugar content in all clones. Principal component analysis based on physiological heat tolerance indexes could clearly distinguish sugarcane genotypes into three heat tolerance clusters. Noteworthy in comparison to the heat-sensitive varieties, sugarcane genotype that possessed higher degrees of heat tolerance Co 99004 displayed higher chlorophyll content, CSI, antioxidant enzyme activities, NR activity, RWC, total phenols, sucrose-metabolizing enzymes, soluble sugar content and leaf gas exchange and lower level of lipid peroxidation and membrane injury index.

## Introduction

Sugarcane is an important industrial crop used for sugar and bio-energy. It is one of the world’s major C4 crops that mainly grow in the tropic and sub-tropic regions. Weather and climate related events (i.e., growth environment of atmospheric [CO_2_], temperature, precipitation, and other extreme weather) are the key factors for sugarcane production worldwide, especially in many developing countries (Zhao and Li 2015). The rise in temperature even by a single degree beyond the threshold level is considered as heat stress in plants (Hasanuzzaman et al. 2013). Increases in temperature may cause yield declines between 2.5% and 10% across a number of agronomic species throughout the twenty-first century (Hatfield et al. 2011). The unfavorable temperature may significantly affect photosynthesis, respiration, water balance, and membrane stability of leaves reported by Kaushal et al. (2016).

Chlorophyll fluorescence (F_v_/F_m_ ratio) has been suggested as quantitative measures of the photochemical efficiency of the PSII complex under different environmental stresses (Adams et al. 1990). Nitrate reductase (NR) involved in nitrogen metabolism, play important role in amino acid biosynthesis, and regulates the protein synthesis (Harris et al. 2000). Proline and soluble sugars is necessary to regulate osmotic activities and maintaining the cell water balance, membrane stability and by buffering the cellular redox potential (Farooq et al. 2008). Secondary metabolites such as phenolics play roles in abiotic stress responses generally associated with tolerance to heat (Wahid 2007). Sucrose synthesis is catalyzed by sucrose phosphate synthase (SPS), sucrose synthase (SS) and its degradation is catalyzed by invertase (Preiss 1982). Gomathi et al. (2017) reported in sugarcane that average reduction of SS and SPS activity were recorded at elevated temperature (42 °C), While in tolerant variety SS and SPS activity was higher under elevated temperature. The ability to sustain leaf gas exchange [net photosynthesis (*Pn*), Stomatal conductance (*gs*), transpiration rate and CO_2_ assimilation rates] under heat stress has a direct relationship with heat tolerance (Hall 1992). Oxidative stress can cause lipid peroxidation and consequently membrane injury, protein degradation, and enzyme inactivation (Meriga et al. 2004). The reactive oxygen species-scavenging enzymes, for example, ascorbic peroxidase, catalase, guaiacol peroxidase and superoxide dismutase are enhanced by heat stress (Chaitanya et al. 2002; Gomathi and Kohila 2016). Heat stress impairs mitochondrial functions thereby resulting in the induction of oxidative damage that manifests in lipid peroxidation, detected by malondialdehyde (MDA) content (Vacca et al. 2004).

Sugarcane production may have been negatively affected and will continue to be considerably affected by increases in the frequency and intensity of extreme environmental conditions due to climate change. The degree of climate change impact on sugarcane is associated with geographic location and adaptive capacity. However, there has been little research conducted to document these effects as found by Kumudini et al. (2014). Based on pot and field studies with intensive measurements of physiological, growth, and yield traits, we also found that some sugarcane genotypes are more tolerant to stress environment than others (Zhao et al. 2015). To our knowledge, heat stress in sugarcane has received much less attention than the other abiotic stresses. Sugarcane varietal evolution in the future requires yield stability even under harsh climates, understanding of the metabolic and molecular signal transcription processes and the interaction to high temperatures is absolutely necessary. Therefore, to screening new sugarcane cultivars tolerant to heat stress that can contribute to adaptation to climate change (especially for elevated CO_2_ and temperature) by discovering physiological screening technologies can mitigate the negative effect of climate change and improve sugarcane yields, productivity, and sustainability.

## Materials and methods

A pot culture experiment (with confirmation trail) was conducted at Plant Physiology Section, Crop Production Division, ICAR-Sugarcane Breeding Institute, Coimbatore for selection of tolerant sugarcane genotype for high-temperature stress during 2016–2018. The seven sugarcane genotypes used in the present study includes five commercial canes (Co 06022, Co 0315, Co 8021, Co 86032 and Co 99004) and two wild sugarcanes (*Spontaneum* Spp.) genotypes (Taiwan -96 and SES -150). Two sets of pot culture experiment were contacted simultaneously, one for formative phase (150 days) and another one grand growth phase (210 days). The experiment laid out Completely Randomized Block Design with replication thrice. Normal recommended agronomic practices were performed for these experiments.

### Heat stress treatment

In order to develop a study more applicable to field conditions, experimentally heat stressed sugarcane genotypes received a temperature 4–5 °C above its optimum temperature range, an increase which corresponds tightly to climate change model predictions. Control plants were grown under optimal conditions at 37/28 ± 2 °C day/night with a 12-h photoperiod. Heat stressed plants were grown at 45/32 ± 2 °C during the day/night with a 12-h photoperiod and for a total of 15 days, with 60–70% relative humidity, and light intensity 395–410 μmol m^−2^ s^−1^.

### Quantitative analysis of pigment content

Chlorophyll content was estimated by Witham et al. (1971) and the amount of chlorophyll content was calculated using the following equations: Chlorophyll ‘a’ = (12.7 × A_663_) − (2.69 × A_645_) × (V/1000 × W), Chlorophyll ‘b’ = (22.9 × A_645_) − (4.68 × A_663_) × (V/1000 × W) and Total chlorophyll = (20.2 × A_645_) + (8.02 × A_663_) × (V/1000 × W). Chlorophyll Stability Index (CSI) was estimated by Koleyoras (1958) and the chlorophyll content variations between the control and treatment were calculated as CSI. SPAD values of leaves were recorded as described by Peng et al. (1993): using the chlorophyll meter (SPAD - 502, Soil Plant analysis Development Section, Minolta Camera Co. Ltd., Japan).

Chlorophyll fluorescence of the leaves was measured using chlorophyll fluorometer OS-30p (Opti-Sciences, Hudson, USA). The ratio F_V_/F_M_ issued to assess the quantum efficiency for photochemistry of PSII (Krause and Weis 1991; Oliveiram et al. 2002). Relative water content (RWC) was measured as described by Barrs and Weatherley (1962). RWC = [(fresh weight − dry weight)/(turgid weight − dry weight)] × 100. Leaf gas-exchange measurements, including the rate of net photosynthesis (An), stomatal conductance (gs), the rate of transpiration (E) and the intercellular CO_2_ concentration (Ci), were made using a portable Li-6400 m (LI-COR Inc., Lincoln, NE, USA).

### Biochemical assays

Nitrate reductase (NR) activity in leaf was done according to the procedure of Hageman and Hucklesby (1971) with slight modifications. The enzyme activity (NR) was expressed as μ mole NO_2_ g^−1^ fw h^−1^. Analysis of proline content was estimated by the modified procedure of Bates et al. (1973). It was estimated with reference to the calibration curve and expressed as µg g^−1^ tissue FW. The total phenol content was determined by Malick and Singh (1980) and the concentration of phenols express as mg phenols/1 g extract. *Estimation of Sucrose*-*metabolizing enzymes:* Sucrose phosphate synthase (SPS) and sucrose synthase (SS) activity were estimated by the method described by Hubbard et al. (1989). Acid and neutral invertases were assayed by Hatch and Glasziou (1963) method. The total soluble sugar was estimated by Anthrone method (DuBois et al. 1956). *Determination of antioxidant enzymes activities:* Superoxide Dismutase was conveniently assayed using a slightly modified procedure (Madamanchi et al. 1994) and originally described by Beauchamp and Fridovich (1971). The enzyme activity is expressed as min^−1^ g^−1^. Calculation: (maximum absorbance − minimum absorbance) × 60 × 2. Peroxidase (POD) activity was estimated by the method of Putter (1974). The level of lipid peroxidation was measured by estimating malondialdehyde (MDA) content according to the method of Heath and Packer (1968). The concentration of MDA was calculated using its extinction coefficient of 155 mm^−1^ cm^−1^. Membrane injury index (MII) was determined by Deshmukh et al. (1991) recording the electrical conductivity of leaf leachates in double distilled water at 40 and 100 °C. MII = (C_1_/C_2_) × 100.

### Statistical analysis

The experiments were arranged in a completely randomized design with three replications. The data obtained were analyzed by ANOVA and all means were separated at the *P* < 0.05 level using the LSD test. All calculations and data analyses were performed using the SPSS 16.0 for Windows software package. All the data obtained were converted to stress tolerance indexes before Pearson’s correlation, principle component analysis (PCA) and cluster analyses. Stress tolerance index was defined as the observed value of a target trait under a given stress level divided by the mean value for that trait under the control (Zeng et al. 2002). Principle component analysis and Cluster analysis were performed using the XLSTAT.

## Results and discussion

For evolving heat stress tolerant sugarcane genotypes, it is necessary to understand the basic information on physiological and metabolic changes and their interaction with genotypes taking place under heat stress condition. Plant responses to high temperatures are mediated by both their inherent ability to survive and their ability to acquire tolerance to heat stress. In the present study, biochemical characterization of five sugarcane genotypes and two *S. spontaneum* spp. were undertaken for differences in their response to heat stresses. Sugarcane crop in the field is frequently subjected to heat stresses that affect adversely their growth, development and productivity.

### Chlorophyll content and stability

The efficacy of light captured to drive photosynthesis is strongly related to the chlorophyll concentration in the leaf. Heat stress had shown the adverse effect on chlorophyll content, chlorophyll stability index (CSI) and SPAD value of sugarcane genotypes at formative phase (FP) and grand growth phase (GGP) are presented in Tables 1 and 2. Under controlled condition, sugarcane genotypes Co 86032 (1.60 mg g^−1^ FW and 81.2) and Co 99004 (1.58 mg g^−1^ FW and 81.0) had highest total chlorophyll content and CSI respectively. In the present study, when the crop was exposed to heat stress at 45 ± 2 °C, a significant decrease in chlorophyll content, CSI and SPAD value were observed in all the genotypes, suggesting structural damage to the chloroplast in sugarcane genotype due to the high-temperature. Under heat stress condition, higher level of total chlorophyll content, CSI and SPAD value were observed in tolerant genotypes Co 99004 (0.87 mg g^−1^ FW, 72.8 and 32.8), SES 150 (0.77 mg g^−1^ FW, 65.5 and 32.6) and Co 06022 (0.76 mg g^−1^ FW, 62.7 and 30.0), respectively, at formative phase. Average decrease over the control was 15.22 and 15.14% for chlorophyll ‘a’, 26.18 and 25.61% for chlorophyll ‘b’ 18.07 and 17.87% for total chlorophyll and 28.0 and 27.5% for CSI and 19.7 and 18.8% for SPAD value at FP and GGP, respectively, due to high temperature stress.

**Table 1:** Changes chlorophyll content and chlorophyll stability index in sugarcane genotype under exposure to heat stress (pooled data)

Sugarcane genotypes/parameters	Chlorophyll ‘a’ (mg g^−1^ Fw)		Chlorophyll ‘b’ (mg g^−1^ Fw)		Total chlorophyll content (mg g^−1^ Fw)		Chlorophyll stability index	
	Control	Heat stress	Control	Heat stress	Control	Heat stress	Control	Heat stress
*Formative phase*								
Co 06022	1.13 ± 0.0057 d	0.98 ± 0.0059 b	0.39 ± 0.0019 c	0.30 ± 0.0021 c	1.52 ± 0.0076 c	1.28 ± 0.0080 b	78.1 ± 0.36 b	62.7 ± 0.29 c
Co 0315	0.98 ± 0.0049 f	0.80 ± 0.0051 e	0.36 ± 0.0018 e	0.24 ± 0.0019 f	1.34 ± 0.0067 e	1.04 ± 0.0071 e	76.9 ± 0.36 c	39.0 ± 0.18 g
Co 8021	1.08 ± 0.0054 e	0.88 ± 0.0057 d	0.37 ± 0.0019 d	0.26 ± 0.0019 e	1.45 ± 0.0072 d	1.14 ± 0.0075 d	77.7 ± 0.36 bc	49.8 ± 0.23 f
Co 86032	1.18 ± 0.0059 a	0.97 ± 0.0062 bc	0.42 ± 0.0021 a	0.29 ± 0.0023 d	1.60 ± 0.0080 a	1.26 ± 0.0085 c	81.2 ± 0.38 a	52.4 ± 0.24 e
Co 99004	1.16 ± 0.0058 b	1.06 ± 0.0060 a	0.42 ± 0.0021 a	0.33 ± 0.0022 a	1.58 ± 0.0079 a	1.39 ± 0.0083 a	81.0 ± 0.37 a	72.8 ± 0.34 a
Taiwan 96	1.14 ± 0.0057 cd	0.96 ± 0.0059 c	0.39 ± 0.0019 c	0.30 ± 0.0021 c	1.53 ± 0.0076 b	1.26 ± 0.0080 c	78.2 ± 0.36 b	54.9 ± 0.25 d
SES 150	1.15 ± 0.0057 bc	0.98 ± 0.0060 b	0.40 ± 0.0020 b	0.31 ± 0.0021 b	1.55 ± 0.0078 bc	1.29 ± 0.0081 b	78.4 ± 0.36 b	65.5 ± 0.30 b
*Grand growth phase*								
Co 06022	1.19 ± 0.0049 c	1.03 ± 0.0051 b	0.41 ± 0.0015 d	0.32 ± 0.0016 b	1.60 ± 0.0064 c	1.35 ± 0.0067 b	84.0 ± 0.39 b	67.8 ± 0.31 c
Co 0315	1.03 ± 0.0040 e	0.84 ± 0.0042 e	0.38 ± 0.0012 e	0.25 ± 0.0013 e	1.41 ± 0.0052 e	1.09 ± 0.0055 e	82.7 ± 0.38 c	42.2 ± 0.19 g
Co 8021	1.13 ± 0.0044 d	0.92 ± 0.0046 d	0.38 ± 0.0013 e	0.28 ± 0.0014 d	1.51 ± 0.0057 d	1.20 ± 0.0060 d	83.6 ± 0.39 bc	54.2 ± 0.25 f
Co 86032	1.24 ± 0.0049 a	1.02 ± 0.0051 b	0.45 ± 0.0015 a	0.31 ± 0.0016 c	1.69 ± 0.0063 a	1.33 ± 0.0066 c	87.4 ± 0.40 a	57.0 ± 0.26 e
Co 99004	1.21 ± 0.0053 b	1.11 ± 0.0056 a	0.44 ± 0.0017 b	0.35 ± 0.0017 a	1.65 ± 0.0070 b	1.46 ± 0.0073 a	87.1 ± 0.40 a	79.0 ± 0.36 a
Taiwan 96	1.19 ± 0.0048 c	1.00 ± 0.0050 c	0.41 ± 0.0015 d	0.32 ± 0.0016 b	1.60 ± 0.0063 c	1.32 ± 0.0066 c	84.1 ± 0.39 b	59.2 ± 0.27 d
SES 150	1.20 ± 0.0049 bc	1.03 ± 0.0051 b	0.42 ± 0.0016 c	0.32 ± 0.0016 b	1.62 ± 0.0065 c	1.35 ± 0.0067 b	84.3 ± 0.39 b	70.7 ± 0.33 b

Values represent the mean ± SE (n = 3). Letters indicate significant differences at *P* < 0.05 using the LSD tests

**Table 2:** Changes SPAD reading, chlorophyll florescence, RWC, NR and proline in sugarcane genotype under exposure to heat stress (pooled data)

Sugarcane genotypes/parameters	SPAD reading		Chlorophyll florescence (F_v_/F_m_ ratio)		Relative water content (RWC)		Nitrate reductase (NR) (μ mol NO_2_ min^−1^ mg^−1^ protein)		Proline content (μ moles g^−1^ Fw)	
	Control	Heat stress	Control	Heat stress	Control	Heat stress	Control	Heat stress	Control	Heat stress
*Formative phase*										
Co 06022	37.0 ± 0.17 c	30.8 ± 0.14 b	0.718 ± 0.0033 ab	0.644 ± 0.0030 b	80.9 ± 0.37 b	70.9 ± 0.33 b	80.1 ± 0.37 b	64.9 ± 0.30 b	13.1 ± 0.061 b	21.4 ± 0.10 c
Co 0315	35.8 ± 0.17 d	24.2 ± 0.11 e	0.707 ± 0.0033 c	0.602 ± 0.0028 e	76.3 ± 0.35 e	58.7 ± 0.27 g	65.1 ± 0.30 e	45.4 ± 0.21 g	10.8 ± 0.050 e	14.6 ± 0.07 f
Co 8021	36.1 ± 0.17 d	26.7 ± 0.12 d	0.711 ± 0.0033 bc	0.615 ± 0.0028 d	76.6 ± 0.35 de	61.6 ± 0.28 f	67.1 ± 0.31 d	47.9 ± 0.22 f	11.0 ± 0.051 f	15.8 ± 0.07 e
Co 86032	38.0 ± 0.18 a	28.9 ± 0.13 c	0.719 ± 0.0033 ab	0.630 ± 0.0029 c	83.4 ± 0.39 a	68.6 ± 0.32 d	87.1 ± 0.40 a	62.9 ± 0.29 c	13.8 ± 0.064 a	19.7 ± 0.09 d
Co 99004	37.1 ± 0.17 bc	32.8 ± 0.15 a	0.721 ± 0.0033 ab	0.652 ± 0.0030 ab	83.0 ± 0.38 a	78.0 ± 0.36 a	87.3 ± 0.40 a	73.3 ± 0.34 a	12.2 ± 0.056 c	25.2 ± 0.12 a
Taiwan 96	37.2 ± 0.17 b	31.2 ± 0.14 b	0.721 ± 0.0033 ab	0.650 ± 0.0030 ab	77.5 ± 0.36 cd	66.5 ± 0.31 e	77.3 ± 0.36 c	56.6 ± 0.26 e	11.6 ± 0.054 e	21.1 ± 0.10 c
SES 150	37.4 ± 0.17 b	32.6 ± 0.15 a	0.727 ± 0.0034 a	0.656 ± 0.0030 a	78.3 ± 0.36 c	69.8 ± 0.32 c	79.1 ± 0.37 b	62.0 ± 0.29 d	11.9 ± 0.055 d	21.9 ± 0.10 b
*Grand growth phase*										
Co 06022	39.7 ± 0.18 b	33.5 ± 0.15 c	0.772 ± 0.0036 ab	0.697 ± 0.0032 c	86.9 ± 0.40 b	76.9 ± 0.36 b	86.9 ± 0.40 b	70.4 ± 0.33 b	14.8 ± 0.068 b	24.1 ± 0.11 c
Co 0315	38.5 ± 0.18 c	26.6 ± 0.12 f	0.761 ± 0.0035 c	0.655 ± 0.0030 f	80.9 ± 0.37 e	63.4 ± 0.29 f	70.7 ± 0.33 f	50.4 ± 0.23 g	12.2 ± 0.057 f	16.8 ± 0.08 f
Co 8021	38.8 ± 0.18 c	28.9 ± 0.13 e	0.765 ± 0.0035 bc	0.667 ± 0.0031 e	81.2 ± 0.38 e	66.9 ± 0.31 e	72.8 ± 0.34 e	53.0 ± 0.24 f	12.3 ± 0.057 f	18.0 ± 0.08 e
Co 86032	40.8 ± 0.19 a	31.6 ± 0.15 d	0.774 ± 0.0036 ab	0.683 ± 0.0032 d	89.5 ± 0.41 a	74.9 ± 0.35 c	93.2 ± 0.43 a	69.4 ± 0.32 c	15.5 ± 0.072 a	22.8 ± 0.11 d
Co 99004	39.9 ± 0.18 b	35.8 ± 0.17 a	0.776 ± 0.0036 ab	0.708 ± 0.0033 a	89.1 ± 0.41 a	85.2 ± 0.39 a	93.2 ± 0.43 a	80.1 ± 0.37 a	13.8 ± 0.064 c	28.9 ± 0.13 a
Taiwan 96	40.0 ± 0.18 b	33.9 ± 0.16 b	0.775 ± 0.0036 ab	0.710 ± 0.0033 ab	82.7 ± 0.38 d	72.0 ± 0.33 d	83.8 ± 0.39 d	61.8 ± 0.29 e	13.0 ± 0.060 e	23.9 ± 0.11 c
SES 150	40.2 ± 0.19 b	35.4 ± 0.16 a	0.782 ± 0.0036 a	0.717 ± 0.0033 a	84.0 ± 0.39 c	75.6 ± 0.35 c	85.8 ± 0.40 c	67.4 ± 0.31 d	13.4 ± 0.062 d	24.7 ± 0.11 b

Values represent the mean ± SE (n = 3). Letters indicate significant differences at *P* < 0.05 using the LSD tests

According to the results of two growth stage of the sugarcane genotypes, the FP was the sensitive phase and reduction percentage was higher compared to the GGP. The above results clearly show that loss of chlorophyll is directly linked with heat stress in sugarcane genotypes. The change in chlorophyll contents was used to evaluate the influence of environmental stress on plant growth and yield. Among the genotypes stress tolerant index of chlorophyll content was higher in Co 99004 at both FP and GGP, respectively (Table 3). In this studies indicated that high chlorophyll concentrations are associated with improved crop yield in tolerant genotypes as reported early research in wheat by Verma et al. (2004). The reduction in chlorophyll content, CSI and SPAD value were found higher in heat susceptible genotypes (Co 0315) as compared to heat stress tolerant. The decrease in chlorophyll ‘a’, chlorophyll ‘b’, total chlorophyll, CSI and SPAD value in response to induced heat stress has also been reported previously by Gosavi et al. (2014) in sorghum, Kumar et al. (2012b) in maize and rice.

**Table 3:** Stress tolerant index (STI) for sugarcane genotype under exposure to heat stress at formative phase (FP) and grand growth phase (GGP) (pooled data)

Sugarcane genotypes/parameters	Co 06022		Co 0315		Co 8021		Co 86032		Co 99004		SES-91		SES-150	
	FP	GGP	FP	GGP	FP	GGP	FP	GGP	FP	GGP	FP	GGP	FP	GGP
Chlorophyll ‘a’	0.86	0.87	0.81	0.82	0.81	0.81	0.82	0.82	0.91	0.92	0.84	0.84	0.85	0.86
Chlorophyll ‘b’	0.76	0.78	0.66	0.67	0.70	0.74	0.69	0.69	0.78	0.80	0.76	0.78	0.77	0.77
Total chlorophyll content	0.84	0.84	0.77	0.77	0.78	0.79	0.78	0.79	0.88	0.88	0.82	0.83	0.83	0.83
Chlorophyll stability index	0.80	0.81	0.51	0.51	0.64	0.65	0.65	0.65	0.90	0.91	0.70	0.70	0.84	0.84
SPAD reading	0.83	0.84	0.68	0.69	0.74	0.75	0.76	0.77	0.88	0.90	0.84	0.85	0.87	0.88
Chlorophyll fluorescence	0.90	0.90	0.85	0.86	0.86	0.87	0.88	0.88	0.90	0.91	0.90	0.92	0.90	0.92
Proline content	1.63	1.63	1.34	1.37	1.43	1.46	1.43	1.47	2.06	2.10	1.82	1.83	1.84	1.84
Relative water content	0.88	0.89	0.77	0.78	0.80	0.82	0.82	0.84	0.94	0.96	0.86	0.87	0.89	0.90
Total phenolics content	1.27	1.28	1.20	1.22	1.23	1.24	1.23	1.24	1.42	1.44	1.20	1.22	1.21	1.21
Superoxide dismutase	0.89	0.90	0.57	0.64	0.68	0.68	0.73	0.77	1.05	1.16	0.76	0.84	1.03	1.01
Peroxidase	0.91	0.93	0.73	0.82	0.84	0.85	0.87	0.88	1.03	1.04	0.76	0.77	0.84	0.85
Lipid peroxidation	1.19	1.18	1.67	1.67	1.39	1.36	1.28	1.27	1.09	1.07	1.08	1.05	1.07	1.04
Membrane injury index	1.12	1.10	1.27	1.25	1.25	1.23	1.25	1.22	1.06	1.05	1.13	1.13	1.03	1.03
Nitrate reductase	0.81	0.81	0.70	0.71	0.71	0.73	0.72	0.74	0.84	0.86	0.73	0.74	0.78	0.79
Sucrose phosphate synthase	0.85	0.87	0.74	0.76	0.75	0.77	0.76	0.78	0.89	0.90	0.85	0.86	0.86	0.87
Sucrose synthase	0.74	0.76	0.66	0.67	0.67	0.69	0.68	0.70	0.78	0.81	0.69	0.72	0.72	0.74
Acid invertase	0.81	0.83	0.63	0.66	0.69	0.72	0.75	0.77	0.89	0.91	0.69	0.73	0.71	0.74
Neutral invertase	0.80	0.82	0.58	0.63	0.60	0.67	0.66	0.70	0.87	0.91	0.75	0.77	0.82	0.85
Soluble sugar content	1.30	1.32	1.10	1.12	1.11	1.13	1.13	1.17	1.36	1.36	1.10	1.11	1.11	1.11
Photosynthetic rate	0.55	0.71	0.43	0.54	0.44	0.54	0.45	0.55	0.60	0.80	0.35	0.53	0.36	0.60
Stomatal conductance	0.89	0.91	0.74	0.78	0.76	0.81	0.77	0.81	0.95	0.97	0.71	0.76	0.77	0.82
Transpiration rate	0.76	0.77	0.70	0.70	0.71	0.71	0.71	0.72	0.83	0.86	0.66	0.67	0.74	0.74
Intercellular CO_2_ concentration	0.81	0.82	0.69	0.71	0.71	0.73	0.73	0.74	0.86	0.88	0.66	0.68	0.71	0.72

Stress tolerance index was defined as the observations under heat stress divided by the means of the controls

### Chlorophyll fluorescence

The ratio of F_v_/F_m_ is an important parameter describing the physiological state of photosynthesis organelle and serve as an indicator showing the activity of photosynthesis through the evaluation of release amount of chlorophyll fluorescence. A significant decreased in chlorophyll fluorescence (F_v_/F_m_ ratio) was observed in sugarcane of all the genotypes subjected to the crop was exposed to heat stress (Table 2). Under heat stress condition, the highest F_v_/F_m_ ratio was observed in tolerant SES 150 (0.656 and 0.717), Co 99004 (0.652 and 0. 708) and Co 06022 (0.644 and 0.697) genotypes at FP and GGP, respectively. Average F_v_/F_m_ ratio decrease over the control was 11.4 and 10.5% at formative and grand growth phase respectively. Among the genotypes stress tolerant index was higher in Co 99004 (0.90 and 0.91) and it range 0.85–0.90 and 0.86–0.92 at FP and GGP, respectively (Table 3). The results obtained in the present investigation are concomitant with the earlier reported by Cui et al. (2006). However, under heat stress, the conduction of PSII electrons is affected so as to lower the ratio of F_v_/F_m_. The reduction in F_v_/F_m_ ratio was mainly due to a decrease in the variable fluorescence at higher temperatures, which could be due to inefficient energy transfer from the light-harvesting Chl a/b complex to the reaction center (Briantais et al. 1986).

### Relative water content (RWC)

Leaf RWC is a reliable indicator of leaf water deficit status at the time of sampling. It is often used to examine the response of a plant stress. Tolerant genotypes of Co 99004, Co 06022 and SES-150 were able to maintain relatively high leaf RWC of 78.0, 70.0 and 69.8, respectively (Table 2), when subjected to heat stress, while sensitive genotype of Co 0315 showed the highest fold decrease of RWC over the control was observed 23.1 and 21.7% at FP and GGP, respectively, compared to rest of the genotypes at FP (Table 4). Similar result was reported in maize by Chen et al. (2012). Average decrease RWC over the control was 14.7 and 13.4% at FP and GGP respectively. The stress tolerance index of RWC at FP and GGP ranged from 0.77 to 0.94 and 0.78–0.96, respectively (Table 3). The decrease RWC in response to induced heat stress has also been reported previously in *Lotus creticus* (Anon et al. 2004) and tomato (Morales et al. 2003).

**Table 4:** Folding % increase/decrease for sugarcane genotype under exposure to heat stress at formative phase (FP) and grand growth phase (GGP) (pooled data)

Sugarcane genotypes/parameters	Co 06022		Co 0315		Co 8021		Co 86032		Co 99004		SES-91		SES-150	
	FP	GGP	FP	GGP	FP	GGP	FP	GGP	FP	GGP	FP	GGP	FP	GGP
Chlorophyll ‘a’	13.3	13.4	18.4	18.4	18.5	18.6	17.8	17.7	8.6	8.3	15.8	16.0	14.8	14.2
Chlorophyll ‘b’	23.1	22.0	33.3	34.2	29.7	26.3	31.0	31.1	21.4	20.5	23.1	22.0	22.5	23.8
Total chlorophyll content	15.8	15.6	22.4	22.7	21.4	20.5	21.3	21.3	12.0	11.5	17.6	17.5	16.8	16.7
Chlorophyll stability index	19.7	19.3	49.3	49.0	36.0	35.2	35.5	34.8	10.1	9.4	29.8	29.7	16.4	16.1
SPAD reading	16.7	15.8	32.4	31.1	26.0	25.4	23.9	22.7	11.6	10.1	16.2	15.2	12.8	11.9
Chlorophyll florescence	10.2	9.8	14.8	13.9	13.6	12.8	12.3	11.7	9.6	8.8	9.9	8.5	9.7	8.3
Proline content	62.8	63.2	34.4	37.4	43.1	46.0	43.3	47.1	105.9	109.7	82.4	83.2	83.5	83.8
Relative water content	12.3	11.4	23.1	21.7	19.6	17.6	17.7	16.3	6.1	4.4	14.1	13.0	10.8	10.0
Total phenolics content	27.2	27.8	20.4	22.3	22.6	23.7	23.3	23.5	42.3	43.7	20.2	21.9	20.8	21.0
Superoxide dismutase	− 10.7	− 9.6	− 42.9	− 36.4	− 31.5	− 31.7	− 26.5	− 22.9	5.3	15.9	− 23.7	− 15.7	3.3	1.0
Peroxidase	− 9.1	− 6.7	− 27.5	− 18.2	− 16.0	− 15.0	− 13.3	− 12.2	3.1	3.7	− 23.9	− 22.9	− 16.3	− 15.5
Lipid peroxidation	18.9	18.2	67.5	66.5	38.9	35.6	27.9	26.5	9.0	7.1	8.4	5.1	7.2	4.3
Membrane injury index	12.1	10.2	27.5	24.8	25.0	23.2	24.8	22.4	6.0	5.3	13.0	12.7	3.4	3.0
Nitrate reductase	19.0	19.0	30.4	28.7	28.6	27.1	27.8	25.6	16.0	14.1	26.8	26.3	21.5	21.4
Sucrose phosphate synthase	15.4	12.6	26.1	24.4	24.8	23.1	24.1	22.2	10.6	10.0	15.0	14.0	14.1	13.4
Sucrose synthase	26.0	23.7	34.0	32.9	33.4	31.2	31.8	29.8	21.8	19.3	30.7	27.6	27.8	26.2
Acid invertase	18.7	17.3	37.1	33.5	30.9	27.7	24.7	22.8	11.4	9.3	30.7	27.4	29.0	26.0
Neutral invertase	20.0	18.5	41.5	37.3	40.0	32.9	34.4	30.4	13.3	8.6	25.5	23.2	17.9	14.7
Soluble sugar content	29.7	31.5	10.2	11.6	11.4	12.7	12.6	16.6	36.2	36.3	10.0	10.9	10.7	11.4
Photosynthetic rate	45.1	28.9	56.5	46.1	55.8	45.6	54.8	45.2	39.8	19.8	65.3	47.1	64.4	39.9
Stomatal conductance	11.1	8.5	25.7	22.0	23.7	18.9	23.2	18.7	4.6	2.9	28.9	24.0	22.6	18.0
Transpiration rate	23.7	23.2	30.2	29.7	28.9	29.1	28.9	27.8	17.4	13.7	33.9	32.5	25.8	25.8
Intercellular CO_2_ concentration	19.2	18.1	30.7	29.3	29.3	27.4	27.4	26.2	14.0	12.1	33.6	31.9	29.3	28.4

### Nitrate reductase (NR)

Nitrate reductase (NR) is the enzyme, which is involved in nitrogen metabolism, play important role in amino acid biosynthesis, and regulates the protein synthesis. NR activity of sugarcane genotypes at FP and GGP was determined and the result obtained is shown in Table 2. In the present study, the variability in terms of NR activity existed at different genotypes under heat stress, the highest NR activity under heat stress condition was observed significantly in tolerant genotypes Co 99004 (73.3 µ mol NO_2_ min^−1^ mg^−1^ protein) and Co 06022 (64.9 µ mol NO_2_ min^−1^ mg^−1^ protein) and while the lowest NR activity was recorded in Co 0315 (45.4 µ mol NO_2_ min^−1^ mg^−1^ protein). The mean NR activity, % fold decreased in over the control was lower in heat tolerant genotypes (Co 99004) 15.95%, (Co 06022) 19.02% and it decreased fold % higher in susceptible genotypes (Co 0315) 30.38% at FP (Table 4). The similar trend was notified at grand growth phase. The average decrease in the control was 24.0 and 22.8% for NR activity at FP and GGP respectively, due to high-temperature stress. Among the genotypes stress tolerant index was higher in Co 99004 (0.84 and 0.86) and it range 0.70–0.84 and 0.71–0.86 at FP and GGP, respectively (Table 3). Hayat et al. (2009) has also reported in mustard that NR activity decreased in heat stressed plants serves as a biochemical adaptation to conserve energy by stopping nitrate assimilation at the initial stage. Haba et al. (2013) also recently stated that the activity of NR decreased in leaves exposed to high temperature in sunflower.

### Proline accumulation

Proline accumulation is another well-known mechanism that has been evolved to cope with heat stress in a number of plant species. In this study, heat stress obviously induced a marked increase in proline accumulation relative to the level of the control (Table 2). It is interesting to note that higher folding % of proline accumulation in stress tolerant sugarcane cultivars of Co 99004 (25.5 µmol g^−1^ fw), SES-150 (21.9 µmol g^−1^ fw) and Co 06022 (21.4 µmol g^−1^ fw) were 106, 83.5 and 62.8% folds over control respectively (Table 4). The lowest proline content was recorded in Co 0315 (17.8 µmol g^−1^ fw) at FP subjected to heat stress and the trend was found to be similar at GGP of the crop. The results obtained in the present investigation are concomitant with the earlier reported by Kumar et al. (2012a, b) in wheat. The stress tolerance index of proline accumulation among the sugarcane cultivars examined. It ranged from 1.34 to 2.06 and 1.37–2.10 at FP and GGP, respectively. The higher stress tolerance index 2.06 and 2.10 was recorded in stress tolerant sugarcane Co 99004 genotype at FP and GGP, respectively (Table 3). Proline was accumulated under heat stress could also act as low mol. Wt. chaperones, stabilizing and protecting the structure of enzymes and proteins, maintaining membrane integrity and scavenging ROS, and a reservoir of nitrogen and carbon source for post stress growth (Hameed et al. 2012).

### Total phenols (TP)

Enhanced synthesis of secondary metabolites under heat stress conditions also protects against oxidative damage. In the present study, the highest accumulation of total phenols (TP) under heat stressed condition was observed in tolerant genotypes Co 99004 and Co 06022 (732 and 636 µg g^−1^ FW), respectively, while the lowest phenols content was recorded in Co 0315 (555 g^−1^ FW) and in both wild sugarcane genotypes at FP (Table 5). Wahid and Ghazanfar (2006) also reported earlier that enhanced synthesis of total phenolics has been directly correlated with heat tolerance of sugarcane. The mean fold increase in TP accumulation over control was higher in stress tolerant Co 99004 (42.3%) followed by Co 06022 (27.2%) and heat stress susceptible Co 0315 (20.4%) (Table 4). The stress tolerance index of total phenols activity at FP and GGP ranged from 1.20 to 1.42 and 1.21–1.44, respectively (Table 3). However, better accumulation of phenolics in tolerant variety may be related to better protection against oxidative damage, screening of harmful radiations, stabilization of sub-cellular structures and improvement in cell water balance as previously reported in *Oenothera biensis* by Fardus et al. (2014).

**Table 5:** Changes total phenolics content, SOD, POD, MII and LP in sugarcane genotype under exposure to heat stress (pooled data)

Sugarcane genotypes/parameters	Total phenolics content (µg g^−1^)		Superoxide dismutase (SOD) (SOD activity units min^−1^ g^−1^ FW)		Peroxidase (POD) (POD activity units per liter)		Membrane injury index (MII)		Lipid peroxidation (nmol malondialdehyde g^−1^ FW)	
	Control	Heat stress	Control	Heat stress	Control	Heat stress	Control	Heat stress	Control	Heat stress
*Formative phase*										
Co 06022	500 ± 2.31 b	636 ± 2.94 b	50.9 ± 0.24 c	45.5 ± 0.21 c	454 ± 2.10 c	413 ± 1.91 b	31.3 ± 0.14 b	35.1 ± 0.16 b	0.95 ± 0.0044 c	1.13 ± 0.0052 b
Co 0315	461 ± 2.13 d	555 ± 2.56 d	35.2 ± 0.16 f	20.1 ± 0.09 g	351 ± 1.62 f	254 ± 1.17 f	56.2 ± 0.26 f	71.7 ± 0.33 f	1.01 ± 0.0047 e	1.69 ± 0.0078 e
Co 8021	490 ± 2.26 c	601 ± 2.78 c	44.8 ± 0.21 e	30.6 ± 0.14 f	404 ± 1.87 e	340 ± 1.57 e	49.4 ± 0.23 e	61.8 ± 0.29 e	0.99 ± 0.0046 d	1.38 ± 0.0064 d
Co 86032	515 ± 2.38 a	635 ± 2.93 b	54.9 ± 0.25 a	40.4 ± 0.19 d	467 ± 2.16 a	405 ± 1.87 c	28.9 ± 0.13 a	36.1 ± 0.17 c	0.93 ± 0.0043 b	1.19 ± 0.0055 c
Co 99004	515 ± 2.38 a	732 ± 3.38 a	54.9 ± 0.25 a	57.8 ± 0.27 a	460 ± 2.13 b	475 ± 2.19 a	29.2 ± 0.13 a	30.9 ± 0.14 a	0.78 ± 0.0036 a	0.85 ± 0.0039 a
Taiwan 96	409 ± 1.89 f	492 ± 2.27 f	45.8 ± 0.21 d	34.9 ± 0.16 e	314 ± 1.45 g	239 ± 1.10 g	39.0 ± 0.18 d	44.0 ± 0.20 d	1.67 ± 0.0077 g	1.81 ± 0.0084 g
SES 150	427 ± 1.97 e	516 ± 2.38 e	52.6 ± 0.24 b	54.4 ± 0.25 b	425 ± 1.96 d	355 ± 1.64 d	35.4 ± 0.16 c	36.5 ± 0.17 c	1.62 ± 0.0075 f	1.73 ± 0.0080 f
*Grand growth phase*										
Co 06022	532 ± 2.46 c	681 ± 3.14 b	55.5 ± 0.26 c	50.2 ± 0.23 c	493 ± 2.28 c	460 ± 2.12 b	28.9 ± 0.13 b	31.8 ± 0.15 b	0.81 ± 0.0037 c	0.96 ± 0.0044 b
Co 0315	490 ± 2.26 e	600 ± 2.77 d	38.4 ± 0.18 f	24.4 ± 0.11 g	380 ± 1.76 f	311 ± 1.44 f	51.8 ± 0.24 f	64.7 ± 0.30 g	0.85 ± 0.0039 d	1.42 ± 0.0066 d
Co 8021	522 ± 2.41 d	645 ± 2.98 c	43.8 ± 0.20 e	29.9 ± 0.14 f	439 ± 2.03 e	373 ± 1.72 e	46.0 ± 0.21 e	56.7 ± 0.26 f	0.85 ± 0.0039 d	1.16 ± 0.0053 c
Co 86032	553 ± 2.55 a	683 ± 3.16 b	59.5 ± 0.27 a	45.8 ± 0.21 d	506 ± 2.34 a	445 ± 2.05 c	26.6 ± 0.12 a	32.6 ± 0.15 c	0.67 ± 0.0031 a	0.85 ± 0.0039 a
Co 99004	542 ± 2.50 b	779 ± 3.60 a	59.3 ± 0.27 ab	68.8 ± 0.32 a	499 ± 2.31 b	518 ± 2.39 a	26.9 ± 0.12 a	28.3 ± 0.13 a	0.79 ± 0.0036 b	0.85 ± 0.0039 a
Taiwan 96	435 ± 2.01 g	530 ± 2.45 f	48.9 ± 0.23 d	41.2 ± 0.19 e	341 ± 1.57 g	263 ± 1.21 g	35.9 ± 0.17 d	40.5 ± 0.19 e	1.44 ± 0.0066 f	1.51 ± 0.0070 f
SES 150	455 ± 2.10 f	550 ± 2.54 e	58.7 ± 0.27 b	59.2 ± 0.27 b	461 ± 2.13 d	389 ± 1.80 d	32.6 ± 0.15 c	33.6 ± 0.15 d	1.39 ± 0.0064 e	1.45 ± 0.0067 e

Values represent the mean ± SE (n = 3). Letters indicate significant differences at *P* < 0.05 using the LSD tests

### Antioxidant enzyme activities

The coordinate function of antioxidant enzymes like Superoxide Dismutase (SOD) and Peroxidase (POD) helps in the processing of reactive oxygen species (ROS) and regeneration of redox ascorbate and glutathione metabolites (Foyer and Nector 2000). In the present study, the heat stressed sugarcane genotypes exhibited a decreased in the activity of SOD and POD over the control in all genotypes, except heat tolerant genotype Co 99004. Under heat stress condition, Co 99004 led to the significantly highest SOD and POD activity of 57.8 and 68.8 Units min^−1^ g^−1^ fw of tissue and 475 and 518 Units per liter at FP and GGP, respectively, suggesting that high temperature could trigger antioxidant enzymes to scavenge ROS to counteract the injurious effect of ROS. Therefore, tolerance to high-temperature stress in crop plants to be associated with an increase in antioxidant activity has been found in agreement with earlier reported in sorghum (Gosavi et al. 2014) and in sugarcane (Gomathi and Kohila 2016). Whereas, SOD and POD activity of Co 0315 susceptible genotype was recorded comparatively less at both stages (Table 5). The ROS activity was found to be higher in Co 99004 under stress (5.3 and 15.9 and 3.1 and 3.7% at FP and GGP, respectively) compared to rest of the genotypes (Table 4) which was reflected in stress tolerance index (Table 3). When ROS increase; chain reactions start in which superoxide dismutase, a metallo-enzyme catalyses the dismutation O_2_^−^ radical to molecular O_2_ and H_2_O_2_ reported in wheat by Kumar et al. (2012a) and peroxidases regulate the relatively stable levels of H_2_O_2_ to water and oxygen molecule reported in Mullberry by Chaitanya et al. (2002).

### Lipid peroxidation (LPO) and membrane injury index (MII)

Lipid peroxidation is a natural metabolic process under normal aerobic conditions and it is one of the most investigated consequences of ROS action on membrane structure and function (Blokhina et al. 2003). Lipid peroxidation is a commonly utilized stress indicator of membrane damage (Taulavuori et al. 2001). In the present study, Lipid peroxidation (LPO) as malondialdehyde (MDA) content 0.85 n mol MDA g^−1^ fw. and membrane injury index (MII) 30.9 were lower under heat stressed condition was observed in tolerant genotype Co 99004, while the highest LPO and MII of was recorded in Taiwan 96, SES-150 and Co 0315 at FP and GGP (Table 5). Earlier researchers reported that the relative tolerance of genotype to heat stress as reflected by its lower LPO, higher membrane stability, maintenance of high f_v_/f_m_ ratio and pigment concentration is related to the levels of activity of its antioxidant enzymes in sugarcane (Abbas et al. 2013). Also, Zhao et al. (2010) found in opium poppy that when the antioxidant enzyme activities were high, MDA content, as well as relative membrane LPO was low. Gomathi et al. (2013) reported in sugarcane that crop exposure to high-temperature caused a significant increase in lipid peroxidation (MDA content) and cell membrane injury. Average LPO and MII increased over the control were 23.1 and 20.4 and 17.4 and 15.8% at FP and GGP respectively, due to high-temperature stress. The stress tolerant index of LPO and MII were higher in heat tolerant genotype of Co 99004 compare to other genotypes (Table 3).

### Sucrose-metabolizing enzymes

Many enzymes in internodes were related to sucrose metabolism, such as invertase, sucrose synthase (SS) and sucrose-phosphate synthase (SPS). Invertases cleave sucrose to glucose and fructose. Sucrose synthase can either cleave sucrose to UDP-glucose and fructose or catalyse the reverse, synthetic reaction. SPS synthesizes sucrose-6-phosphate reported in sugarcane by Gayler and Glasziou (1972). High temperature stress altered the activities of sucrose-metabolizing enzymes (SPS, SS, AI and NI) in sugarcane genotypes. When the crop were exposed to heat stress at 45 ± 2 °C, a significant decrease in sucrose-metabolizing enzymes were observed in all genotypes (Table 6). Heat stress tolerant genotypes had significantly highest activity of sucrose-metabolizing enzymes were observed in tolerant Co 99004 (29.8, 31.1, 27.7 and 36.3 µ mol g fr wt^−1^ h^−1^) and followed by Co 06022 (26.3, 27.8, 24.0 and 30.6 µ mol g fr wt^−1^ h^−1^) genotypes at FP as compared to susceptible genotypes, respectively. The similar trend was observed in GGP. Average decrease over the control was 18.7 and 17.2% for SPS, 29.2 and 27.1% for SS, 25.7 and 23.1% for AI and 27.7 and 23.6% for NI at FP and GGP respectively, due to high temperature stress. The maximum reduction in sucrose-metabolizing enzymes on account of heat stress was observed in Co 0315 and wild genotypes (Table 4). The higher stress tolerance index of SPS, SS, AI and NI (0.89, 0.78, 0.89 and 0.87) were recorded in tolerant variety Co 99004 at FP, respectively (Table 3). Miguel et al. (2007) reported in tomato that the ability of plants to synthesize and accumulate sucrose in leaves under environmental stress is mainly determined by the concerted action of sucrose metabolizing enzymes. At low concentrations sucrose acts as signaling molecule while it has been suggested that in high concentrations it becomes an ROS scavenger reported in *Arabidopsis* by Sugio et al. (2009). However, Ebrahim et al. (1998) also thought the activities of sucrose-metabolizing enzymes decreased in sugarcane leaves under high-temperature stress accompanied with the sucrose content reduced.

**Table 6:** Changes SPS, SS, AI, NI and soluble sugar content in sugarcane genotype under exposure to heat stress (pooled data)

Sugarcane genotypes/parameters	Sucrose phosphate synthase (SPS) (µ mol g fr wt^−1^ h^−1^)		Sucrose synthase (SS) (µ mol g fr wt^−1^ h^−1^)		Acid invertase (AI) (µ mol g fr wt^−1^ h^−1^)		Neutral invertase (NI) (µ mol g fr wt^−1^ h^−1^)		Total Soluble Sugar content (µg g^−1^)	
	Control	Heat stress	Control	Heat stress	Control	Heat stress	Control	Heat stress	Control	Heat stress
*Formative phase*										
Co 06022	31.1 ± 0.14 d	26.3 ± 0.12 b	37.6 ± 0.17 c	27.8 ± 0.13 b	29.5 ± 0.14 c	24.0 ± 0.11 c	38.3 ± 0.18 c	30.6 ± 0.14 b	63.8 ± 0.29 b	82.7 ± 0.38 b
Co 0315	30.8 ± 0.14 d	22.8 ± 0.11 e	33.4 ± 0.15 e	22.1 ± 0.10 e	28.4 ± 0.13 d	17.8 ± 0.08 f	34.9 ± 0.16 e	20.4 ± 0.09 e	50.8 ± 0.23 d	56.0 ± 0.26 e
Co 8021	31.7 ± 0.15 c	23.9 ± 0.11 d	36.6 ± 0.17 d	24.4 ± 0.11 c	29.2 ± 0.13 c	20.2 ± 0.09 d	37.8 ± 0.17 d	22.7 ± 0.10 d	57.6 ± 0.27 c	64.2 ± 0.30 d
Co 86032	34.3 ± 0.16 a	26.0 ± 0.12 bc	40.8 ± 0.19 a	27.8 ± 0.13 b	35.4 ± 0.16 a	26.7 ± 0.12 b	46.4 ± 0.21 a	30.4 ± 0.14 b	71.0 ± 0.33 a	80.0 ± 0.37 c
Co 99004	33.3 ± 0.15 b	29.8 ± 0.14 a	39.8 ± 0.18 b	31.1 ± 0.14 a	31.2 ± 0.14 b	27.7 ± 0.13 a	41.9 ± 0.19 b	36.3 ± 0.17 a	71.0 ± 0.33 a	96.7 ± 0.45 a
Taiwan 96	26.8 ± 0.12 f	22.8 ± 0.11 e	31.1 ± 0.14 e	21.6 ± 0.10 f	25.4 ± 0.12 f	17.6 ± 0.08 f	24.0 ± 0.11 g	17.9 ± 0.08 f	46.2 ± 0.21 f	50.8 ± 0.23 g
SES 150	30.0 ± 0.14 e	25.8 ± 0.12 c	33.1 ± 0.15 f	23.9 ± 0.11 d	27.1 ± 0.13 e	19.2 ± 0.09 e	32.1 ± 0.15 f	26.3 ± 0.12 c	49.7 ± 0.23 e	55.0 ± 0.25 f
*Grand growth phase*										
Co 06022	33.7 ± 0.16 d	29.5 ± 0.14 b	40.8 ± 0.19 c	31.1 ± 0.14 b	32.0 ± 0.15 c	26.5 ± 0.12 c	41.1 ± 0.19 c	33.5 ± 0.15 c	66.6 ± 0.31 c	87.6 ± 0.40 b
Co 0315	33.4 ± 0.15 d	25.3 ± 0.12 f	36.3 ± 0.17 e	24.3 ± 0.11 f	30.1 ± 0.14 d	20.0 ± 0.09 f	37.9 ± 0.17 d	23.7 ± 0.11 f	53.1 ± 0.25 e	59.2 ± 0.27 e
Co 8021	34.4 ± 0.16 c	26.4 ± 0.12 e	39.7 ± 0.18 d	27.3 ± 0.13 d	31.7 ± 0.15 c	22.9 ± 0.11 d	40.8 ± 0.19 c	27.4 ± 0.13 e	60.2 ± 0.28 d	67.8 ± 0.31 d
Co 86032	37.2 ± 0.17 a	28.9 ± 0.13 c	43.7 ± 0.20 a	30.7 ± 0.14 c	38.2 ± 0.18 a	29.5 ± 0.14 b	49.3 ± 0.23 a	34.3 ± 0.16 b	73.1 ± 0.34 b	85.2 ± 0.39 c
Co 99004	36.1 ± 0.17 b	32.5 ± 0.15 a	43.2 ± 0.20 b	34.8 ± 0.16 a	33.9 ± 0.16 b	30.7 ± 0.14 a	46.7 ± 0.22 b	42.7 ± 0.20 a	75.2 ± 0.35 a	102.4 ± 0.47 a
Taiwan 96	29.1 ± 0.13 f	25.0 ± 0.12 f	33.8 ± 0.16 f	24.5 ± 0.11 f	27.6 ± 0.13 f	20.0 ± 0.09 f	26.0 ± 0.12 f	20.0 ± 0.09 g	48.2 ± 0.22 g	53.5 ± 0.25 g
SES 150	32.6 ± 0.15 e	28.2 ± 0.13 d	35.9 ± 0.17 e	26.5 ± 0.12 e	29.4 ± 0.14 e	21.7 ± 0.10 e	34.8 ± 0.16 e	29.7 ± 0.14 d	51.9 ± 0.24 f	57.8 ± 0.27 f

Values represent the mean ± SE (n = 3). Letters indicate significant differences at *P* < 0.05 using the LSD tests

### Total soluble sugar content (TSS)

Total soluble sugars were increased under heat stress for oxidative adjustment. Data herein in Table 6 showed that all studied sugarcane genotypes TSS varied significantly between 46.2 and 71.0 µg g^−1^ fw in non-stressed plant, while heat stress accumulated sugar contents under stress condition ranging from 50.8 to 96.7 µg g^−1^ fw at FP. It was noticed that total sugar content was enhanced under heat stress condition in sugarcane genotypes, maximum and minimum folding % increment were observed in Co 99004 (36.0%) and Co 0315 (10.0%), respectively (Table 4). Theses increases in total sugars in the tolerant genotypes may be due to inhibition of sucrose synthase or invertase activities as reported by Mohamed and Abdel-Hamid (2013) in cotton. In present study, heat stress showed an average increase of 18.4 and 19.9% for total sugars content at FP and GGP respectively. The higher stress tolerance index of 1.36 was recorded in tolerant variety Co 99004 at FP (Table 3). Under stress situation, TSS content was comparatively higher at GGP compared to FP. Hassanein et al. (2012) also reported in fenugreek that the increase in TSS may be acting as an adaptive mechanism for exerting protective effects under heat stress.

### Leaf gas exchange

Leaf gas exchange is considered as one of the indicators to evaluate plants ability under different environment stress condition. Leaf gas exchange measurements including photosynthesis rate (A_n_), stomatal conductance (g_s_), transpiration rate (T) and intercellular CO_2_ concentration (C_i_) were observed on sugarcane genotypes. In the present study, irrespective of varieties and wild species clones, when plant was exposed to heat stress a notable reduction in leaf gas exchange was observed over the control (Table 7). Under heat stress condition, significantly highest photosynthesis rate (A_n_) (11.44 μ mol CO_2_ m^−2^ s^−1^), stomatal conductance (g_s_) (1.30 mol H_2_O m^−2^ s^−1^), transpiration rate (T) (10.83 mmol H_2_O m^−2^ s^−1^) and intercellular CO_2_ concentration (C_i_) (320 µ mol CO_2_m^−2^ s^−1^) were observed in stress tolerant Co 99004 followed by Co 06022 and Co 86032 genotypes, respectively, and the maximum reduction in leaf gas exchange on account of heat stress was observed in Co 0315 at FP. The similar trend was observed in GGP. The average decrease over the control was 52.6 and 37.1% for photosynthesis rate, 19.1 and 15.2% for stomatal conductance, 26.7 and 25.7% for transpiration rate and 25.9 and 26.4% for intercellular CO_2_ concentration at FP and GGP respectively. Among the varieties, Co 99004 attained the highest stress tolerant index (Table 3) at both FP and GGP. Unlike other environmental stresses, in the present study varieties which transpire more water under elevated temperature condition could maintain transpiration cooling and RWC and their by higher photosynthetic rate compared less transpiring varieties. However, some earlier researchers reported that high temperature stress reduces net photosynthetic rate, stomatal conductance in sunflower (Haba et al. 2013), and transpiration of water and CO_2_ diffusion into the leaf tissues in rice (Sikuku et al. 2010). The results of two growths stage of the sugarcane genotypes, FP was sensitive stage and reduction percentage of leaf gas exchange was higher compared to GGP.

**Table 7:** Changes leaf gas exchange parameters in sugarcane genotype under exposure to heat stress (pooled data)

Sugarcane genotypes/parameters	Photosynthetic rate (μ mol CO_2_ m^−2^ s^−1^)		Stomatal conductance (mol H_2_O m^−2^ s^−1^)		Transpiration rate (mmol H_2_O m^−2^ s^−1^)		Intercellular CO_2_ concentration (µ mol CO_2_ m^−2^ s^−1^)	
	Control	Heat stress	Control	Heat stress	Control	Heat stress	Control	Heat stress
*Formative phase*								
Co 06022	13.74 ± 0.063 c	7.55 ± 0.035 b	1.25 ± 0.006 c	1.11 ± 0.005 b	11.75 ± 0.054 c	8.96 ± 0.041 b	330 ± 1.52 c	266 ± 1.23 c
Co 0315	10.41 ± 0.048 e	4.52 ± 0.021 e	0.97 ± 0.004 e	0.72 ± 0.003 e	10.68 ± 0.049 e	7.45 ± 0.034 d	312 ± 1.44 e	216 ± 1.00 e
Co 8021	12.41 ± 0.057 d	5.48 ± 0.025 d	1.06 ± 0.005 d	0.81 ± 0.004 d	11.43 ± 0.053 d	8.13 ± 0.038 c	322 ± 1.49 d	228 ± 1.05 d
Co 86032	15.59 ± 0.072 b	7.05 ± 0.033 c	1.41 ± 0.006 a	1.08 ± 0.005 c	12.47 ± 0.058 b	8.87 ± 0.041 b	384 ± 1.77 a	279 ± 1.29 b
Co 99004	19.01 ± 0.088 a	11.44 ± 0.053 a	1.37 ± 0.006 b	1.30 ± 0.006 a	13.12 ± 0.061 a	10.83 ± 0.050 a	372 ± 1.72 b	320 ± 1.48 a
Taiwan 96	9.37 ± 0.043 f	3.25 ± 0.015 g	0.93 ± 0.004 f	0.66 ± 0.003 f	10.89 ± 0.050 f	7.20 ± 0.033 e	310 ± 1.43 e	206 ± 0.95 f
SES 150	9.48 ± 0.044 f	3.38 ± 0.016 f	0.94 ± 0.004 f	0.73 ± 0.003 e	10.98 ± 0.051 e	8.14 ± 0.038 c	309 ± 1.43 e	219 ± 1.01 e
*Grand growth phase*								
Co 06022	21.8 ± 0.10 c	15.5 ± 0.072 b	1.52 ± 0.007 b	1.39 ± 0.006 b	13.64 ± 0.063 b	10.48 ± 0.048 b	348 ± 1.61 c	285 ± 1.32 c
Co 0315	17.0 ± 0.08 e	9.2 ± 0.042 e	1.15 ± 0.005 d	0.90 ± 0.004 d	12.30 ± 0.057 d	8.65 ± 0.040 d	328 ± 1.51 e	232 ± 1.07 e
Co 8021	19.7 ± 0.09 d	10.7 ± 0.050 d	1.26 ± 0.006 c	1.03 ± 0.005 c	13.22 ± 0.061 c	9.38 ± 0.043 c	336 ± 1.55 d	244 ± 1.13 d
Co 86032	24.8 ± 0.11 b	13.6 ± 0.063 c	1.69 ± 0.008 a	1.38 ± 0.006 b	14.48 ± 0.067 a	10.45 ± 0.048 b	397 ± 1.83 a	293 ± 1.35 b
Co 99004	30.2 ± 0.14 a	24.2 ± 0.112 a	1.71 ± 0.008 a	1.66 ± 0.008 a	14.65 ± 0.068 a	12.65 ± 0.058 a	385 ± 1.78 b	338 ± 1.56 a
Taiwan 96	14.3 ± 0.07 f	7.6 ± 0.035 f	1.08 ± 0.005 f	0.82 ± 0.004 e	12.23 ± 0.057 d	8.25 ± 0.038 e	319 ± 1.47 f	217 ± 1.00 f
SES 150	15.0 ± 0.07 e	9.0 ± 0.042 e	1.11 ± 0.005 e	0.91 ± 0.004 d	11.72 ± 0.054 e	8.70 ± 0.040 d	322 ± 1.49 f	231 ± 1.07 e

Values represent the mean ± SE (n = 3). Letters indicate significant differences at *P* < 0.05 according to Duncan’s multiple range tests

### Principle component (PC) analysis

**Fig. 1:**
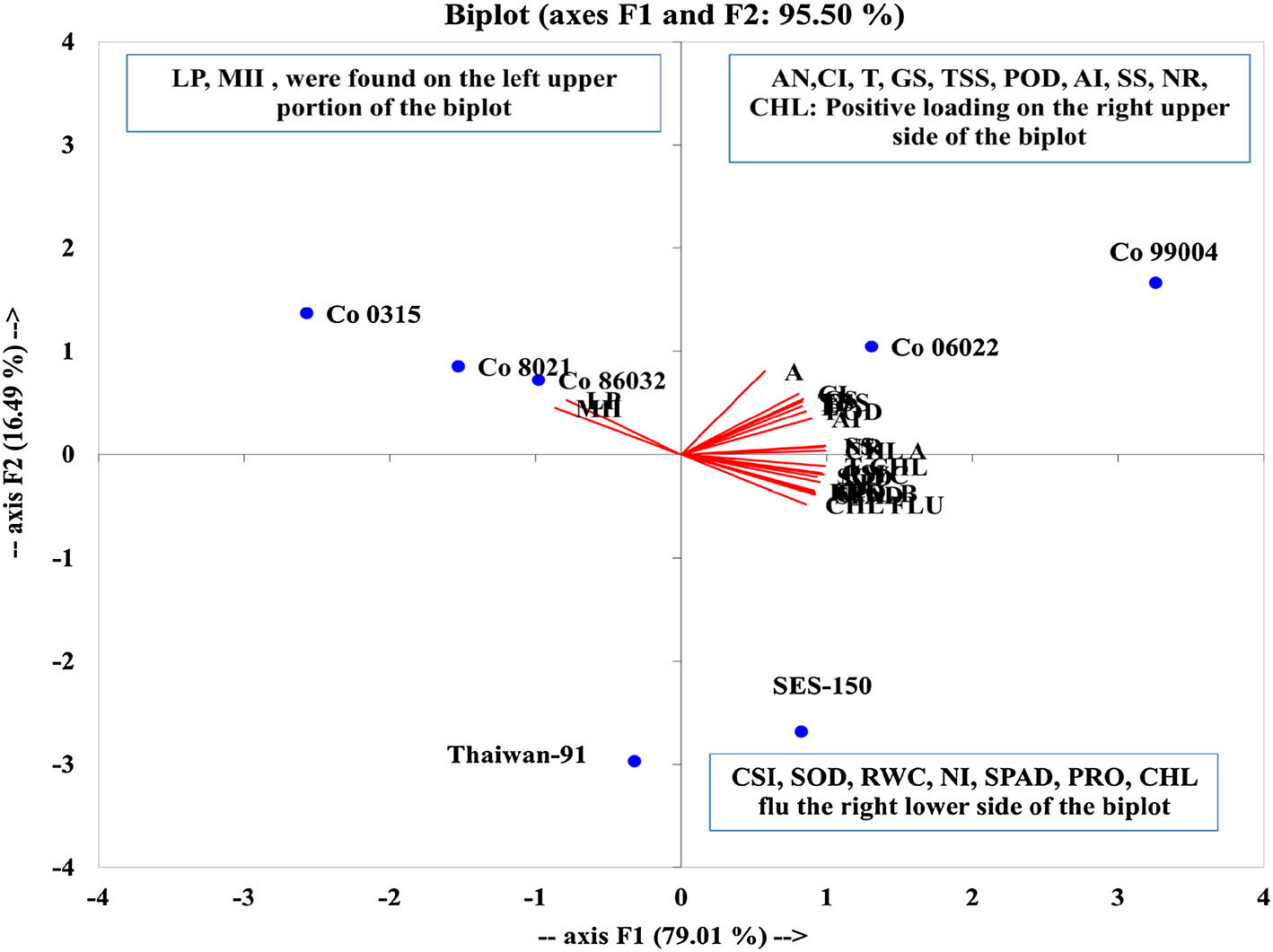
Loading plots of principle components 1 and 2 of the PCA results obtained from physiological data of seven sugarcane cultivars subjected to heat stress

Loading plots of principle component 1 and 2 analysis obtained from physiological data of seven sugarcane genotypes subjected to heat stress are illustrated in Fig. 1. PCA in the current study allowed for easy visualization of complex data and the physiological parameters among seven sugarcane genotypes were separated by PC1 and PC2. In this study, principle component 1 (PC1) describes 79.01% of the original information and principal component 2 (PC2) describes 16.49%. The cumulative percentage of PC1 and PC2 was 95.50% (Fig. 1). To investigate the contributors to the principle component, the physiological loadings in PC1 and PC2 were compared. It was clear that the, AN, CI, T, GS, TSS, POD, AI, SS, NR and CHL A were grouped together with positive loading on the right upper side of the biplot, suggesting that these parameters had a high positive correlation among themselves. Total CHL, CSI, SOD, RWC, NI, CHL B, SPAD, SPS, PRO and CHL FLU were observed on the right lower side of the biplot signifying that these parameters had a positive correlation among themselves. While LP and MII were found on the left upper portion of the biplot suggesting that these parameters had a highly negative and significant correlation among themselves.

Among the seven genotypes, Co 99004 and Co 06022 were grouped together with positive loading on the right upper side of the biplot, suggesting that this genotype found to tolerant with high-temperature stress. The species SES-150 is being grouped right lower portion of the biplot, indicating moderately tolerant to heat stress. While, Co 0315 Co 8021 and Co 86032 were grouped in a left upper portion of the biplot, and Taiwan-96 left lower portion of the biplot suggesting that these genotypes were sensitive to heat stress.

### Hierarchical cluster analysis (HCA)

**Fig. 2:**
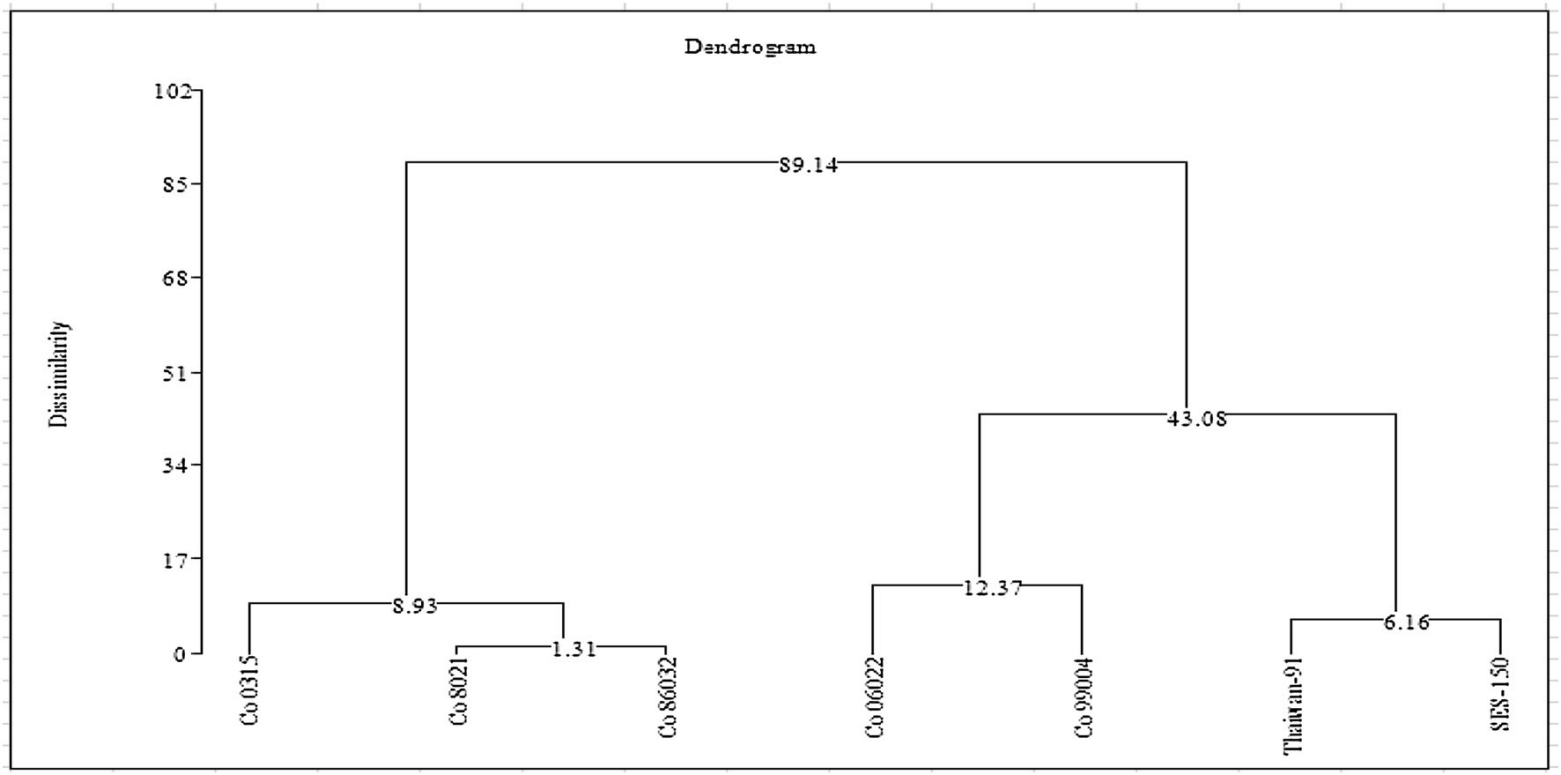
Cluster analysis of the seven sugarcane genotypes based on physiological parameters in heat stress condition

Hierarchical cluster analysis (HCA) was applied to search for classifiers (Fig. 2). The seven sugarcane cultivars were classified into three main clusters. Cluster I represented the heat sensitive group, with considered Co 0315, Co 8021 and Co 86032. Among the heat sensitive genotypes, Co 0315 similarity with 8.93 to other heat sensitive genotypes. Co 8031 with similar with 1.31 to Co 86032. Cluster II represented that heat tolerant group, with considered Co 99004 and Co 06022. Co 06022 similar to Co 99004 with 12.37 similarities. Cluster III represented the heat tolerant as wild sugarcane genotype, with considered SES-150 and Taiwan 96. Cluster II, 46.08 similarities with Cluster III and Cluster I, 89.14 similarities with cluster II and III. The higher similarity distance represents that the higher variation between the tolerant and sensitive genotypes.

## Conclusion

In conclusion, high-temperature stress induced significant physiological and metabolic changes in all sugarcane genotypes at two stages of crop, however formative phase was found to more sensitive to high temperature as compared to grand growth phase. This study showed that physiological parameters such as chlorophyll content, CSI, antioxidant enzymes, enzymes of sucrose metabolism, soluble sugar content, proline content, total phenolics and leaf gas exchange parameters could be used as supplementary or alternative indicators for heat tolerance in sugarcane. Among the genotypes studied, the Co 99004 was found to be highly thermotolerant, as indicated by PCA and cluster analysis, which can be used as donor genotype for high-temperature tolerance. The results also suggest that the identified physiological traits can be used as heat tolerance index for screening larger population for thermotolerance.
